# The development of direct 3-dimensional printing of patient-specific mitral valve in soft material for simulation and procedural planning

**DOI:** 10.1016/j.xjtc.2024.06.008

**Published:** 2024-06-21

**Authors:** Shokoufeh Cheheili Sobbi, Milou Pauli, Marvin Fillet, Jos G. Maessen, Peyman Sardari Nia

**Affiliations:** a Department of Cardiothoracic Surgery, Heart and Vascular Centre Maastricht University Medical Centre, Maastricht, The Netherlands; b Cardiovascular Research Institute Maastricht (CARIM), Maastricht University, Maastricht, The Netherlands; c Department of Biomechanical Engineering, University of Twente, Enschede, The Netherlands

**Keywords:** mitral valve, mitral valve repair, simulation

## Abstract

**Objectives:**

Replicating 3-dimensional prints of patient-specific mitral valves in soft materials is a cumbersome and time-consuming process. The aim of this study was to develop a method for a direct 3-dimensional printing of patient-specific mitral valves in soft material for simulation-based training and procedural planning.

**Methods:**

A process was developed based on data acquisition using 3-dimensional transesophageal echocardiography Cartesian Digital Imaging and Communication of Medicine format, image processing using software (Vesalius3D, Blender, Meshlab, Atum3D Operation Station), and 3-dimensional printing using digital light processing, an additive manufacturing process based on photopolymer resins. Experiments involved adjustment of 3 variables: curing times, model thinness, and lattice structuring during the printing process. Printed models were evaluated for suitability in physical simulation by an experienced mitral valve surgeon.

**Results:**

Direct 3-dimensional printing of a patient's mitral valve in soft material was completed within a range of 1.5 to 4.5 hours. Prints with postcuring times of 5, 7, 10, and 15 minutes resulted in increased stiffness. The mitral valves with 2.0-mm and 2.4-mm thinner leaflets felt more flexible without tear of the sutures through the material. The addition of lattice structures made the prints more compliant and better supported suturing.

**Conclusions:**

Direct 3-dimensional printing of a realistic and flexible patient-specific mitral valve was achieved within a few hours. A combination of thinner leaflets, reduced curing time, and lattice structures enabled the creation of a realistic patient-specific mitral valve in soft material for physical simulation.

## Graphical Abstract

**Figure:**
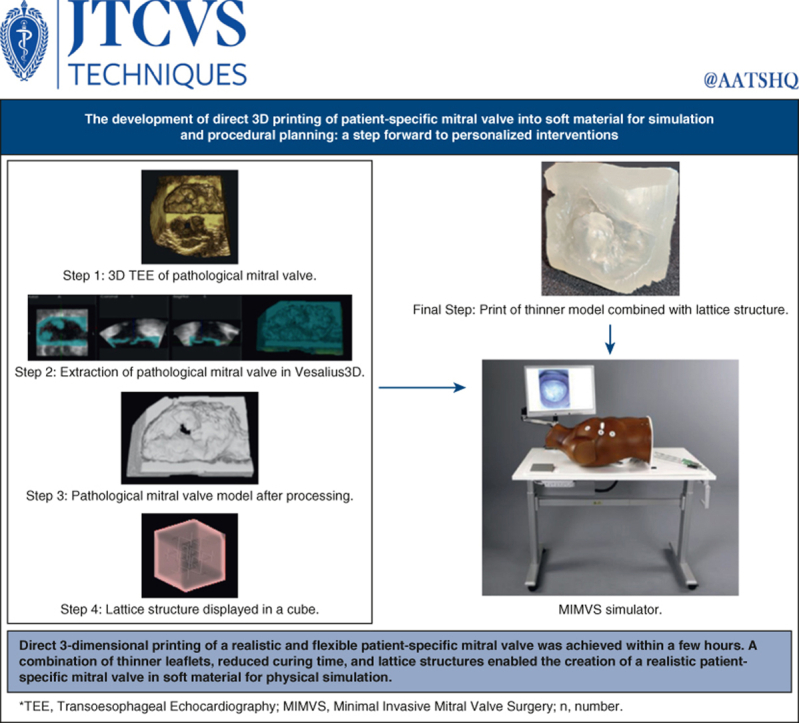

**Figure:**
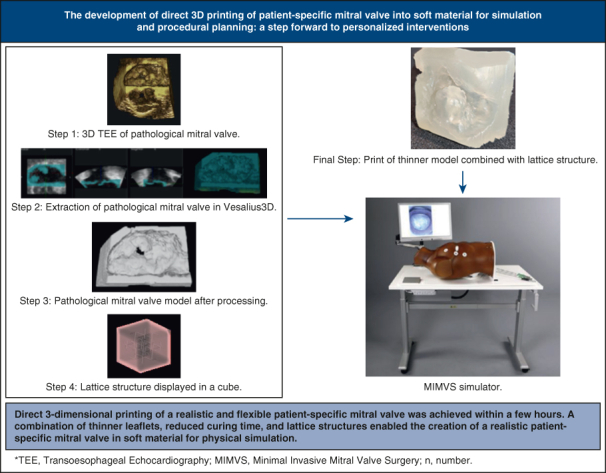
The development of direct 3D printing of patient-specific mitral valve into the soft material for simulation and procedural planning.

**Central Message**

Direct 3D printing of a realistic and flexible patient-specific mitral valve was achieved within a few hours for physical simulation.

**Perspective**

Our developed direct 3D printing method offers a faster means of obtaining patient-specific mitral valves for procedural simulation compared with silicone-casted mitral valves. Mitral valve leaflets, annulus, and the walls of the 3D-printed mitral valve are reported to be suitable for surgical and transcatheter simulator purposes, according to an experienced mitral valve surgeon.

The application of 3-dimensional (3D) printing in procedural planning for many surgical fields has been rapidly expanding over the past few years.^1^, ^2^, ^3^ The use of 3D printing in cardiac surgery could be a valuable method for assessing complex anatomy. This approach greatly benefits peri-interventional decision-making, as well as facilitating guidance of interventional strategy, such as device sizing. It serves as a means of predicting and subsequently reducing the incidence of adverse events.^3^, ^4^, ^5^, ^6^ Mitral valve repair is considered the gold standard for addressing degenerative mitral regurgitation.^7^ However, mitral valve repair in cases involving complex anatomy can be particularly challenging.^8^ Preprocedural physical simulation of patient-specific mitral valve repair could be beneficial in simulation-based training and procedural planning. The high-fidelity minimally invasive mitral valve surgery (MIMVS) simulator developed by Sardari Nia and colleagues has demonstrated excellence as a tool for simulation-based training.^9^^,^^10^ Nonetheless, the replication of 3D prints of patient-specific mitral valves, as used in simulators like the MIMVS simulator, currently involves a cumbersome and time-consuming process of negative mold fabrication and silicone casting, which can take up to a few days. The entire production process may span up to 7 days.^11^ The primary aim of this study was to develop a method for direct 3D printing of patient-specific mitral valves using soft material in a cost-effective production process, specifically designed for simulation-based training and procedural planning.

## Material and Methods

We developed a process composed of 3 steps: data acquisition using 3D transesophageal echocardiography (TEE) Cartesian Digital Imaging and Communication of Medicine format, image processing using software (Vesalius3D, Blender, Meshlab, Atum3D Operation Station), and 3D printing using digital light processing, an additive manufacturing process based on photopolymer resins.

### Requirements

Several requirements were predefined for the creation of realistic patient-specific 3D printed mitral valves (Table E1).

### Data Acquisition and Processing

**Figure 1:**
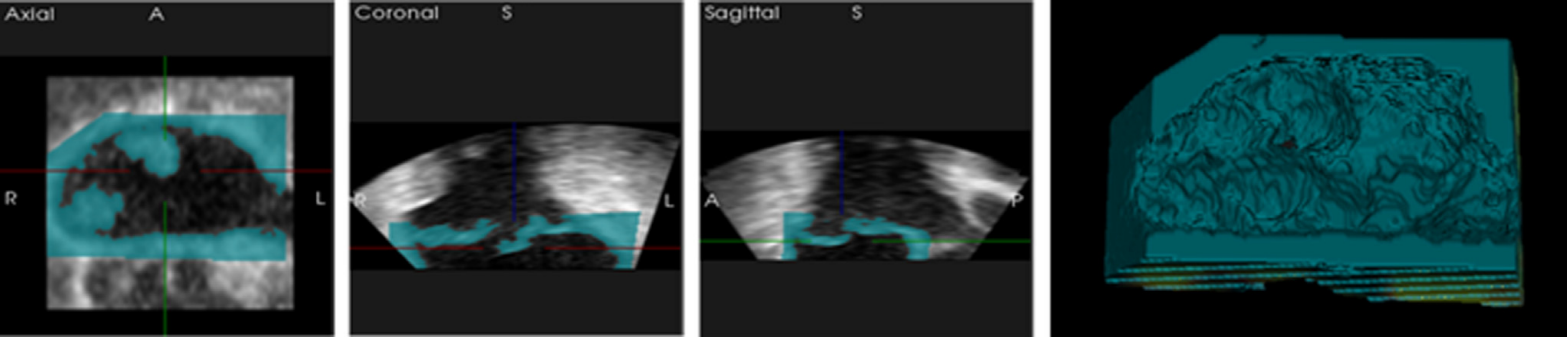
Extraction of pathological mitral valve in Vesalius3D.

A 3D TEE of a Barlow's mitral valve with anterior (A) A2, A3, and posterior (P) P2 and P3 prolapse was used. The images were acquired by an imaging cardiologist specialized in mitral valve. The 3D Cartesian Digital Imaging and Communication of Medicine format was imported into the image processing software Vesalius3D (PS-Medtech) to make an extraction of the valve during the systolic phase of the cardiac cycle (Figure 1). Only open-source software was considered for further processing of the models. The automatic determined thresholds in Vesalius3D were adjusted. The lower threshold remained the same, while the upper threshold was increased to fully extract the mitral valve leaflets and annulus. The region of interest was separated using scissors, erase, and region growing functions and noise, such as blood, were removed. The detailed surface model was exported to an Surface Tessalation Language file (.stl), which is suitable for 3D printing. This file was imported in Meshlab (Istituto di Scienza e Tecnologie dell’Informazione), where the mesh was simplified using a quadric based edge-collapse strategy.^12^ Vertices and faces were reduced with a target number of faces or percentage reduction to make further modifications computationally less intensive. A mesh with half the number of vertices and faces was created. Additionally, Laplacian Smooth averaged each vertex position with weighted positions of neighbor vertices.^13^ Final adjustments, such as local sculpting and noise removal were made in the open-source 3D computer graphics software tool Blender (Blender Foundation). The stl file with the pathological mitral valve was imported into the Atum3D Operation Station for preparation of the print job. The placement and orientation of the model on the building platform can be adjusted, and support structures can be generated with MAGS AI technology or manually. Manual placement was chosen because the MAGS AI technology placed an unnecessary amount of supports. Overhangs were visually evaluated using the slicer print preview for complementary judgment.

### Material and 3D Printing

All 3D prints were created by using the digital light processing station 5-405 with most suitable resin for the direct 3D printing mitral valve application at the time of our research.^14^ A urethane photopolymer with a high softness (Shore 40A) and tear strength (9 N/mm)^15^ was chosen. The prints were cleaned with isopropyl alcohol and further cleaned for an additional 2 minutes in an ultrazone cleaner. After 2 hours of drying, the prints were postcured in a vacuum with Atum3D's postcuring station.^16^

To develop a direct 3D printing process in suitable soft material, we conducted experiments involving adjustments of 3 variables: curing times, thinner models, and building lattice structuring during printing process. The prints and their suitability for physical simulation were evaluated by an experienced mitral valve surgeon.

### Curing Times

**Figure 2:**
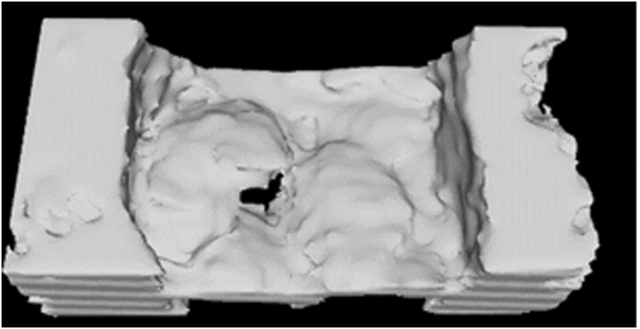
Smaller prints of mitral valve that includes the pathological part of the mitral valve leaflets and parts of the walls on 2 sides.

For optimal material properties, the print should undergo postcuring. Postcuring times were evaluated at 5, 7, 10, and 15 minutes. The pathologic mitral valve model was modified to create smaller prints, which still included the pathological sections of the mitral valve leaflets and portions of the adjacent walls (Figure 2). The printing process remained consistent. Additionally, another print was produced with a curing time reduced by 10% per single layer compared with the default settings. This adjustment was made to reduce adhesion and facilitate easier cleaning.

All the prints were assessed for their stiffness and suture strength. Stiffness was evaluated by an experienced mitral valve surgeon, and suture strength was assessed through suturing.

### Thinner Model

**Figure 3:**
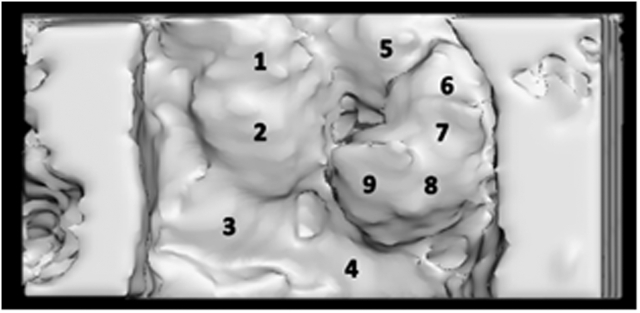
Locations for the thickness measurements.

To achieve a more realistic representation of the mitral valve, thinner leaflets were printed to investigate their impact on flexibility and suture strength. We used the open-source software Meshmixer (Autodesk) to create an offset and an absolute shrinkage of the model in 3 dimensions. We measured various offset distances to obtain printable mitral valve models with offset distances of −1.0 and −1.2 mm. Figure 3 and Table 1 show the thickness measurements at different locations of the model as determined using Meshmixer. After creating the offset, we removed artifacts using Meshmixer and Blender and applied smoothing to certain parts of the model. Additionally, measurements were conducted in Meshmixer based on the data in Figure 3. The thinner models were printed as previously described and postcured for 10 minutes, based on the results of earlier postcuring time tests.

**Table 1:** Measured thicknesses at different locations after correction: A reference model and offset distances of −1.0 and −1.2

Offset distance (mm)	Measured thickness at different locations (mm)								
	1	2	3	4	5	6	7	8	9
0.0 (reference)	5.0	3.7	3.1	5.4	4.4	4.3	3.6	3.5	3.5
−1.0	2.5	1.9	1.4	2.9	2.4	1.3	1.5	1.5	1.6
−1.2	1.8	1.3	1.0	2.7	2.1	0.8	1.1	1.1	1.1

As previously mentioned, an experienced mitral valve surgeon assessed the flexibility of the prints and suture strength.

### Lattice Structures

To achieve a more compliant texture of the atrial walls and the ventricular walls, lattice structures were introduced. We created 5 prints of a section of these walls, each varying in wall thicknesses (in mm), lattice element dimensions (in mm), and lattice element spacing (in mm), as specified in Table 2. Additionally, another print was created by combining lattice structures in the walls with the thinner leaflets, achieved by introducing an offset distance of −1.2 mm. To do this, the leaflets were first separated from the rest of the model and then recombined after lattice structure creation. Meshmixer was used to generate the lattice structures. The walls were isolated from the rest of the model, hollowed out, resulting in separate inner and outer objects. The normals of the inner object were inverted, and a lattice structure was subsequently applied.

**Table 2:** Parameters for creating lattice structures for 5 prints and a model with thinner leaflets

3D print thickness at different locations of mitral valve	Hollow wall thickness (mm)	Lattice element dimension (mm)	Lattice element spacing (mm)
Models	1.0	0.5	1.5
A	0.3	0.3	2.5
B	0.5	0.5	2.0
C	0.5	0.5	1.0
D	1.0	0.5	1.5
E	1.0	1.0	1.5

**Figure 4:**
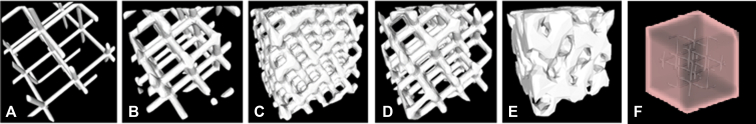
A-E, Lattice structures of the 5 prints displayed in 6 × 6 × 6-mm cubes. F, Lattice structure A displayed in a cube.

The lattice orientation was adjusted by rotating it by +45° along the x-, y-, and z-axes to create a stable and printable structure. Afterward, the lattice was subtracted from the inner object, and following the normal flipping, it was combined with the outer object. Figure 4 provides a representation of the lattice structure parameters within a 6 × 6 × 6-mm cube and also illustrates the lattice structure in combination with an outer shell.

Each print included 2 drainage holes with a diameter of 1.4 mm, placed on the side near the building platform of the 3D printer. In the case of the larger model, there were 4 holes in total, with 2 on each side of the leaflets. These drainage holes allowed any uncured resin to drain out of the model. In some prints, there remained some uncured resin, which was manually removed as much as possible.

Additionally, there was some isopropyl alcohol left inside the prints after cleaning. The isopropyl alcohol was removed in the same manner as the uncured resin.

An experienced mitral valve surgeon assessed the tactile properties and suture strength of the prints.

## Results

Pathological mitral valves were 3D printed with different stiffness levels and subjected to varying postcuring times. Prints postcured for 5, 7, 10, and 15 minutes exhibited negligible differences in material stiffness. However, the material's flexibility still did not meet the desired criteria. To address this, we explored various offset distances, resulting in printable mitral valve models with −1.0 mm and −1.2 mm offsets, effectively reducing the valve's thickness by 2.0 mm and 2.4 mm, respectively. Additionally, the removal of artifacts led to slight variations in the model's thickness compared with the reference, causing minor deviations.

**Figure 5:**
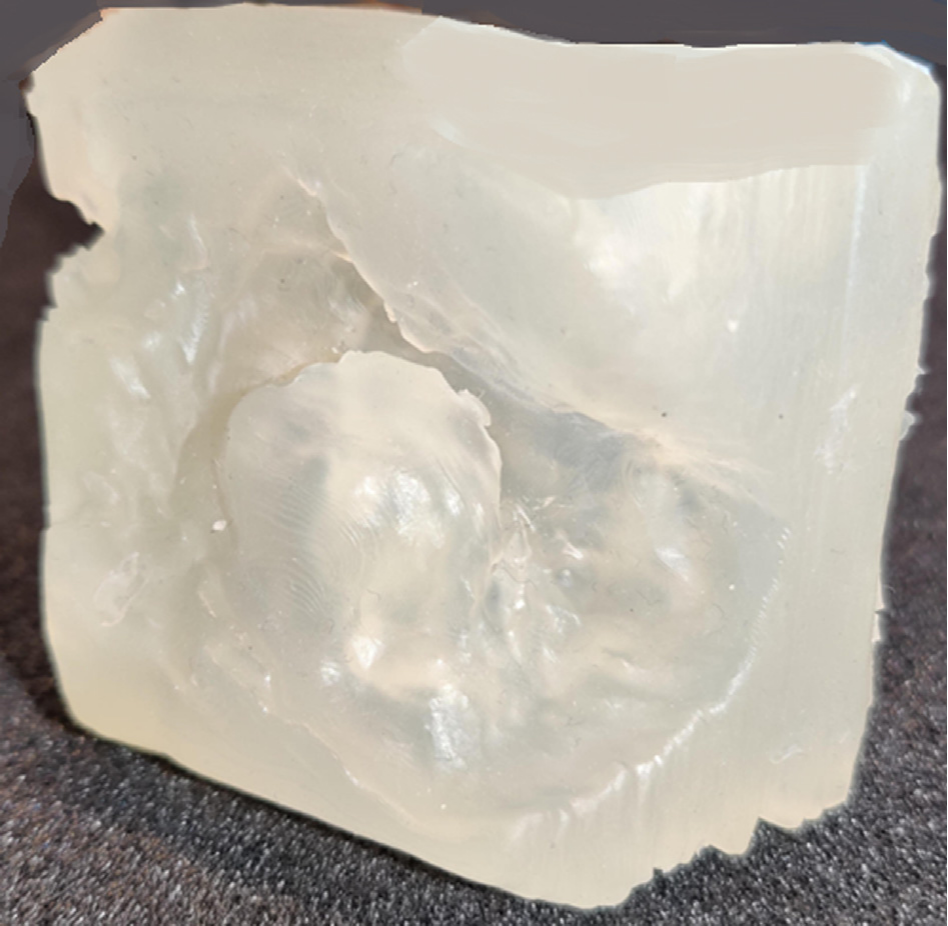
Print of thinner model combined with lattice structure D.

The thinner models successfully replicated the tactile properties of peroperative mitral valves with Print A appearing collapsed. Furthermore, in models C and E, the lattice structures were not fully visible due to accumulated resin between them. Print B exhibited the highest flexibility, but an experienced mitral valve surgeon determined that Print D was the most suitable for simulating real-world scenarios, as sutures did not tear through the material during traction. Lattice structure D was combined with the 2.4-mm thinner mitral valve leaflets, resulting in a printable model that combined the properties of the thinner leaflets with those of the lattice structures (Figure 5).

## Discussion

**Figure 6:**
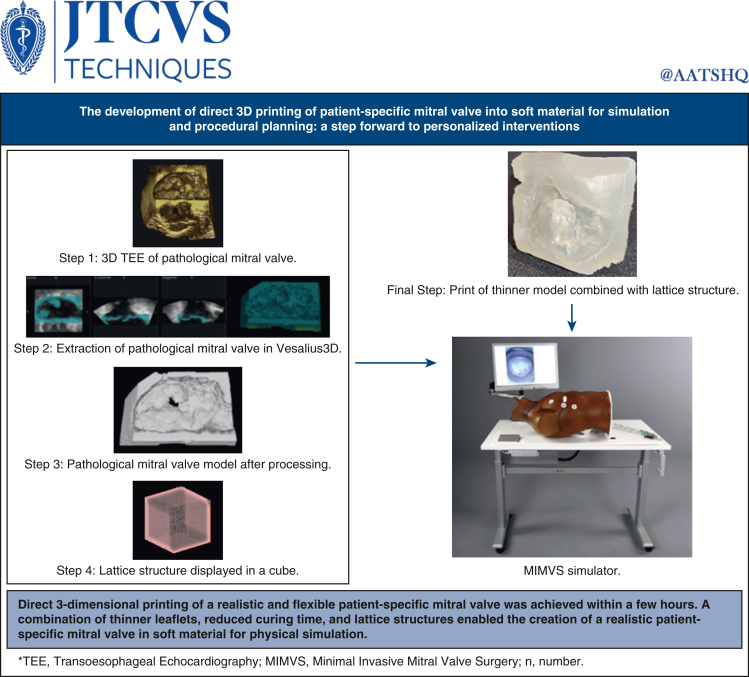
Graphical abstract.

The process of direct 3D printing of the mitral valve, developed in this study (Figure 6), takes between 1.5 and 4.5 hours, depending on the print orientation. Furthermore, multiple mitral valves can be printed simultaneously without increasing the printing time. These prints are suitable for simulation-based purposes because thinner leaflets are more flexible, providing a more realistic feel, and the material does not tear when sutures are applied. However, the technique for creating thinner models needs adjustment because the offset leads to a uniform shrinkage in the model's x, y, and z dimensions, resulting in a smaller model and a deviation from the anatomic structure. Shrinkage should occur only in the intended dimension while preserving the mitral valve's anatomy. Lattice structures significantly influence the perceived stiffness of the prints. Prints featuring lattice structures exhibit greater influence on the perceived stiffness of the prints. Prints featuring lattice structures exhibit greater compliance compared with solid prints. The specific lattice structures used play a crucial role in determining the print properties.^17^, ^18^, ^19^, ^20^

Earlier research indicates that TangoPlus FullCure 930 and Acrylonitrile butadiene styrene are the most commonly used materials for printing mitral valve structures. However, because of the challenges of direct 3D printing with flexible materials, often the printed casts and molds are made from other materials. These molds are subsequently coated or dipped in silicone to achieve flexible valves with more accurate tissue properties.^21^ A challenge with this method is the need for either removable silicone from the cast or mold after hardening or printing the cast or mold in a dissolvable material. As a result, molding requires more time and labor compared with directly printed models. Although molds are commonly 3D printed with patient-specific properties, they do not match the anatomic precision found in directly 3D printed mitral replicas. Additionally, silicone valve models do not incorporate the subvalvular apparatus.

Multi-material 3D printing, involving materials with multiple properties, has been shown in previous studies to partially replicate the anatomic structures and mechanical characteristics of pathological conditions of mitral leaflets to a certain extent.^22^, ^23^, ^24^ However, these methods are not suitable for directly 3D printing a mitral valve.

In our previous study, we successfully simulated the prospective surgical repair of a P2 prolapse using a deformable patient-specific mitral replica derived from 3D TEE within our MIMVS simulator. Subsequently, we performed a real-life surgical repair, achieving success without any adjustments to the simulated surgical technique.^11^ This underscores the potential value of integrating physical 3D modeling with preprocedural imaging in intraprocedural decision-making. Therefore, using directly 3D printed patient-specific mitral valves in soft materials could offer significant benefits for pre- and intraprocedural decision-making and training.

Vukicevic and colleagues^22^^,^^23^ used direct printing, using multi-material elastomeric TangoPlus materials from Stratasys (Eden Prairie), which were then compared with freshly harvested porcine leaflet tissue. Notably, all TangoPlus varieties exhibited lower stiffness than the maximum tensile elastic modulus of porcine mitral valve tissue. However, the mesh creation process involved substantial manual input, and the final rapid prototyping models did not incorporate chordae tendineae.^22^, ^23^, ^24^ Engelhardt and colleagues^24^ used a technique to fabricate patient-specific silicone mitral valve phantoms, incorporating both the papillary muscles and the chordae tendinae. Nevertheless, the relative softness of the silicone material prevented a perfect replication of the strength of the subvalvular apparatus.^22^, ^23^, ^24^

### Study Limitations

Several limitations of our study should be discussed. In our study, we did not print the subvalvular apparatus, such as chordae tendineae and papillary muscles, because the morphology of these structures could not be completely identified by TEE. Prior investigations demonstrate that these structures could be identified using cardiac computed tomography (CT)-derived data; however, it requires 12 to 20 hours of meticulous work.^22^, ^23^, ^24^ Furthermore, the leaflets cannot be fully retrieved from CT scans. The combination of CT and TEE images could provide a complete 3D reconstruction of mitral apparatus. However the objective of this study was to test the proof-of-concept of direct 3D printing. This was done only for the leaflets, annulus, and walls, because these were the limiting steps for 3D printing.

Additionally, in this study the valves were printed in systole to capture the pathology and the repair in the simulator was done by correcting the coaptation gap to more than 8 mm coaptation using chordal or resection techniques (simulator has set of disposable papillary muscles) and using a proper annuloplasty. The absence of the subvalvular apparatus makes the direct 3D-printed models in our study less suitable for the prospective surgical repair of patient-specific mitral valve involving complex anatomy and testing the repair result. Future research on the direct 3D printing of patient-specific mitral valves, using a combination of TEE and CT-derived data, is recommended.

Another limitation is that the direct 3D prints of the mitral valve in our study were evaluated by a single mitral valve surgeon, and their properties were not objectively assessed. To assess stiffness, compression tests could be performed, and the tactile qualities of the prints should undergo blind evaluation by multiple mitral valve surgeons. The evaluation criteria should prioritize the realistic feel of the 3D prints. Furthermore, the ability to withstand suture tension could be assessed by measuring the force required to tear the suture through the material when all sutures have the same width and depth. Additionally, the maximum force during suturing could be measured in a simulated setting involving multiple surgeons. The 3D printed mitral valve should demonstrate resistance to these forces.

Moreover, the clinical value of preoperative practice with patient-specific 3D printed mitral valves should be studied in a randomized control trial, where one of the outcome measures is the rate of mitral valve repairs.

## Conclusions

Our developed direct 3D printing method offers a faster means of obtaining patient-specific mitral valves for procedural simulation compared with silicone-casted mitral valves. Mitral valve leaflets, annulus, and the walls of the 3D-printed mitral valve are reported to be suitable for surgical and transcatheter simulator purposes, according to an experienced mitral valve surgeon. Furthermore, the material does not tear during suturing. However, further research is needed to objectively assess and study the clinical added value of direct 3D printing of patient-specific mitral valves.

### Webcast

You can watch a Webcast of this AATS meeting presentation by going to: https://www.aats.org/resources/the-development-of-direct-3-d-print-of-patient-specific-mitral-valve-for-a-high-fidelity-minimally-invasive-mitral-valve-surgery-simulator-a-step-forward-to-personalized-surgery.

**Figure:**
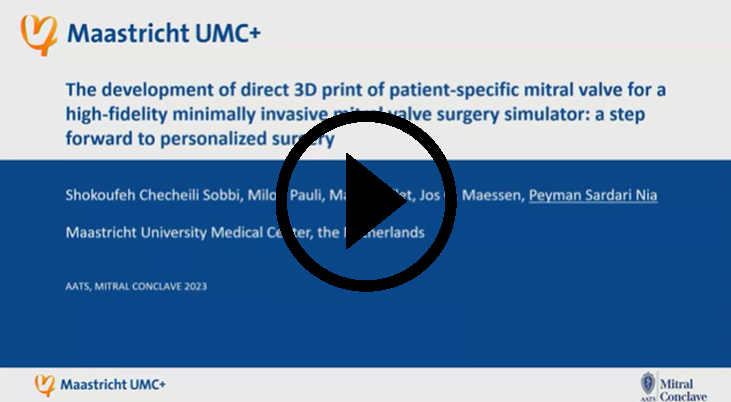

## Conflict of Interest Statement

P.S.N. is the inventor of the Minimally Invasive Mitral Valve simulator that is commercialized by Simurghy and reports consultancy agreements with NeoChord, Edwards Lifesciences, Medtronic, Abbott, and Fujifilm. All other authors reported no conflicts of interest.

The *Journal* policy requires editors and reviewers to disclose conflicts of interest and to decline handling or reviewing manuscripts for which they may have a conflict of interest. The editors and reviewers of this article have no conflicts of interest.

## Figures and Tables

**Table E1:** Requirements for patient-specific 3D printed mitral valves

Requirements
The mitral valve should have a realistic feeling to mitral valve surgeons.
Ability to pull on a suture without tearing the material (referred as “suture strength”).
Anatomy of the mitral valve should match with the perioperative anatomy of the mitral valve.
The mitral valve model should be suitable with the already existing MIMVS simulator.
The lead time of the mitral valve should be faster than the mitral valve lead time of silicone casted mitral valve. Preferably 1 day.

*MIMVS*, Minimally invasive mitral valve surgery.
